# Genome Sequences of Multidrug-Resistant *Salmonella enterica* subsp. *enterica* Serovar Infantis Strains from Broiler Chicks in Hungary

**DOI:** 10.1128/genomeA.01400-16

**Published:** 2016-12-15

**Authors:** Tímea Wilk, Móni Szabó, Ama Szmolka, János Kiss, Endre Barta, Tibor Nagy, Ferenc Olasz, Béla Nagy

**Affiliations:** a Agricultural Biotechnology Institute of the National Agricultural Research and Innovation Centre, Gödöllő, Hungary; b Institute for Veterinary Medical Research, Centre for Agricultural Research, Hungarian Academy of Sciences, Budapest, Hungary

## Abstract

Three strains of *Salmonella enterica* serovar Infantis isolated from healthy broiler chickens from 2012 to 2013 have been sequenced. Comparison of these and previously published *S*. Infantis genome sequences of broiler origin in 1996 and 2004 will provide new insight into the genome evolution and recent spread of *S*. Infantis in poultry.

## GENOME ANNOUNCEMENT

*Salmonella enterica* serovar Infantis is the most commonly reported serovar in broilers in Europe also representing the fourth most widespread serovar isolated from human cases (1). Here, we present the genome sequences of three recent strains of the prevalent Hungarian clone B of *S*. Infantis isolated from broilers in 2012 and 2013 that represent new antibiotic resistance and plasmid profiles compared to the strains reported earlier (2–4).

From total DNA of strains SI3337/12, SI757/13, and SI786/13, 600 to 630 bp fragment libraries were prepared by UD GenoMED (Debrecen, Hungary) and 2 × 300 bp Illumina paired-end genome sequencing was performed by the University of Szeged, Department of Biochemistry and Molecular Biology (Szeged, Hungary) as a custom service using Illumina MiSeq platform. The read counts were 1.6 million for SI3337/12, 4.5 million for SI757/13 and 2.9 million for SI786/13 and the estimated coverage of the genomes was 95×, 287×, and 178×, respectively.

The reads were *de novo* assembled using A5-miseq (5) and the genome sequences were annotated using the RAST annotation server (6). The lengths of chromosomal contigs of strains SI3337/12, SI757/13, and SI786/13 were determined as 4,652,703, 4,656,322, and 4,656,981 bp, respectively. We set the taxon to *Salmonella enterica*, the genetic code to *11* (*Archaea*, bacteria) and obtained 4,670, 4,682, and 4,674 annotated genes; 221, 223, and 220 tRNA genes; 51, 52, and 54 rRNA genes; and 52.4%, 51.2%, and 51.5% G+C content for SI3337/12, SI757/13, and SI786/13, respectively.

Pairwise comparison (7) of the chromosomal nucleotide sequences revealed striking similarity (99.99%) of the three strains. The genomes of SI3337/12, SI757/13, and SI786/13 strains showed 99.98%, 99.91%, and 99.99% similarity to the previously sequenced strains SI69/94 (GenBank accession no. NZ_JRXB00000000), 1326/1328 (GenBank accession no. LN649235), and SI54/04 (GenBank accession no. NZ_JRXC00000000) (4).

The draft sequences of all three strains contain some scaffolds that cannot be aligned to the genome of the earliest Hungarian isolate SI69/94, where the full draft sequence was determined. Additionally, the genome analysis confirmed the presence of large plasmids in all three *S*. Infantis strains as follows: ca. 277 kbp and a 45 kbp plasmid in SI3337/12, a 277 kbp plasmid in SI757/13, and a 210 kbp plasmid in SI786/13.

Evaluation of genomic data of these and previously sequenced *S*. Infantis strains of poultry and human origin reveals evolutionary events that might contribute to faster spread of these *S*. Infantis clones in poultry (2–4) and in humans (8), but also indicates different clonality of the *S*. Infantis strains isolated from exotic animals of unrelated geographic areas (9).

### Accession number(s).

The draft genome sequences of SI3337/12, SI757/13, and SI786/13 have been deposited in the NCBI GenBank database under the accession numbers MIJS00000000, MIJT00000000, and MIJR00000000, respectively.
